# *Coxiella burnetii* Femoro-Popliteal Bypass Infection: A Case Report

**DOI:** 10.3390/microorganisms11092146

**Published:** 2023-08-24

**Authors:** Farah Azouzi, Louis Olagne, Sophie Edouard, Serge Cammilleri, Pierre-Edouard Magnan, Pierre-Edouard Fournier, Matthieu Million

**Affiliations:** 1 Laboratoire de Microbiologie CHU Sahloul Sousse Tunisie, LR20SP06, Faculté de Médecine de Sousse Tunisie, Université de Sousse, Sousse 4003, Tunisia; azouzi.farah@gmail.com; 2 Service de Médecine Interne, Centre Hospitalier Universitaire Gabriel-Montpied, 63000 Clermont-Ferrand, France; lolagne@chu-clermontferrand.fr; 3 UMR MEPHI, Institut Hospitalo-Universitaire Méditerranée Infection, Institut de la Recherche pour le Développement, Assistance Publique-Hôpitaux de Marseille, Aix-Marseille Université, 13005 Marseille, France; sophie.edouard@ap-hm.fr; 4 French Reference Center for Rickettsioses, Q Fever and Bartonelloses, Institut Hospitalo-Universitaire Méditerranée Infection, 13005 Marseille, France; pierre-edouard.fournier@univ-amu.fr; 5 Service de Médecine Nucléaire Hôpital de la Timone, Assistance Publique-Hôpitaux de Marseille, 13005 Marseille, France; serge.cammilleri@ap-hm.fr; 6 Service de Chirurgie Vasculaire, Hôpital Timone, Assistance Publique-Hôpitaux de Marseille, 13385 Marseille, France; pierre-edouard.magnan@ap-hm.fr; 7 UMR VITROME, Institut Hospitalo-Universitaire Méditerranée-Infection, Institut de la Recherche pour le Développement, Service de Santé des Armées, Assistance Publique-Hôpitaux de Marseille, Aix-Marseille Université, 13005 Marseille, France

**Keywords:** Q fever, *Coxiella burnetii*, PET-scan, vascular graft infection

## Abstract

Cardiovascular infections are the most severe and potentially lethal among the persistent focalized *Coxiella burnetii* infections. While aortic infections on aneurysms or prostheses are well-known, with specific complications (risk of fatal rupture), new non-aortic vascular infections are increasingly being described thanks to the emerging use of 18-fluorodeoxyglucose positron emission tomography (^18^F-FDG PET-scan). Here, we describe an infection of a femoro-popliteal bypass that would not have been diagnosed without the use of PET-scan. It is well-known that vascular prosthetic material is a site favorable for bacterial persistence, but the description of unusual anatomical sites, outside the heart or aorta, should raise the clinicians’ awareness and generalize the indications for PET-scan, with careful inclusion of the upper and lower limbs (not included in PET-scan for cancer), particularly in the presence of vascular prostheses. Future studies will be needed to precisely determine their optimal management.

## 1. Introduction

Q fever is caused by *Coxiella burnetii*, an obligate intracellular bacterium that has been reported in almost every country worldwide. Human contamination often occurs after the inhalation of infected aerosols. Clinical presentation of acute Q fever is nonspecific and may be confused with flu-like syndrome. Patients infected with *Coxiella burnetii* may develop persistent focalized infections, especially if they have pre-existing cardiovascular lesions such as aneurysms, vascular grafts, and valvular defects. This second phase of the disease may remain asymptomatic until revealed by further complications [1,2]. Vascular graft infections are rarer but life-threatening and challenge physicians because of relapse risk and complications such as vascular rupture that can lead to death [3]. We report here the first case, to our knowledge, of *C. burnetii* femoro-popliteal bypass infection [1,4].

## 2. Case Presentation

An 81-year-old man with a history of severe Parkinson’s disease, deep vein thrombosis and pulmonary embolism, was admitted to Clermont-Ferrand Hospital for recurrent fever evolving for 4 months.

He had a history of chronic obliterative arterial disease of the lower limbs, right leg ischemia, and bilateral popliteal aneurysms operated with a bypass installation (bilateral venous femoral popliteal bypass) in 2000. In 2001, he underwent revision surgery for exclusion of an aneurysm at the end of the right superficial femoral artery, complicated by distal emboli requiring amputation of the second and third toe of the right foot.

In March 2016, he presented with a relapsing fever. A blood screening (June 2016) showed a C reactive protein level of 70 mg/L. Blood cultures were negative. The Doppler ultrasound (June 2016) showed a permeable bypass, and a bilateral popliteal aneurysm’s thrombosis, compressive on the popliteal veins. He had a cure of the aneurysm in October 2016.

Q fever serology (July 2016) was positive using the indirect immunofluorescence assay (IF) with antibody titers to phase I IgG, IgM, and IgA of 1:800, 0, and 1:100, respectively, and to phase II IgG, IgM, and IgA of 1:200, 0, and 0, respectively [5]. Specific real-time polymerase chain reactions (PCR) for *C. burnetii* (targeting the IS1111 and IS30A repeated insertion sequences) on blood and serum were negative [5]. IgGs to cardiolipin were negative (<20 GPL/mL).

The PET-scan (July 2016) showed diffuse aortic and arterial hypermetabolism: a discrete fixation of the walls of the aortic arch and a pronounced hypermetabolism on the wall of the left popliteal aneurysm and on the right femoral artery. Transthoracic echocardiography showed no valvulopathies. Nevertheless, *Coxiella burnetii* endocarditis remained possible since the PET-scan also showed fixation of the mitral valve in addition to a pulmonary node.

No source of exposure to *Coxiella burnetii* infection was found for this patient, although his vascular condition was a risk factor of persistent focalized infection. Based on the serological result and the pronounced hypermetabolism of the femoro-popliteal bypass, the diagnosis of *C. burnetii* persistent vascular infection was retained and the patient was treated with doxycycline (200 mg per day) and hydroxychloroquine (200 mg three times a day) with clinical improvement.

Due to the patient’s precarious condition, surgery was not performed and thus no microbiological analysis of the bypass could be conducted, but PET-scan combined with *C. burnetii* serology were strong criteria for the diagnosis.

**Figure 1:**
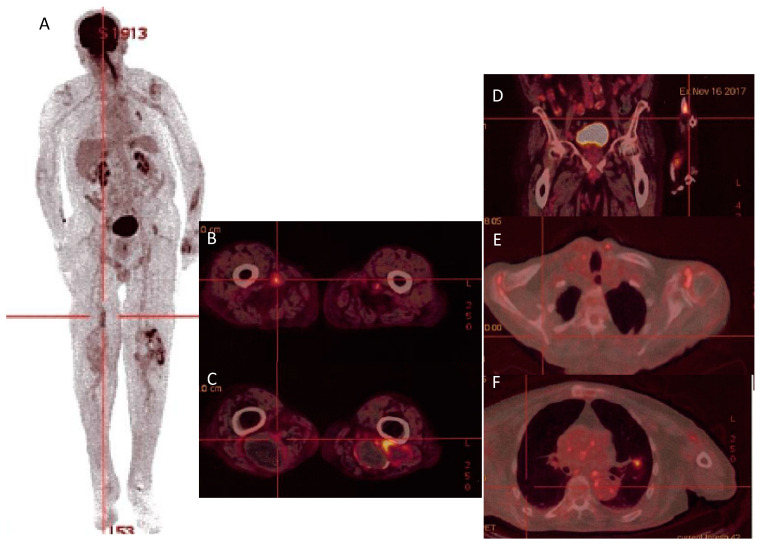
PET-scan (16 November 2017) showing hypermetabolism of the left radius (**A**), left and right popliteal aneurysms (**B**), left femoropopliteal bypass (**C**), the aorta, bilateral inguinal adenopathies (**D**), shoulders (**E**), left pulmonary nodule, and mitral valve (**F**).

Therapeutic drug monitoring of doxycycline and hydroxychloroquine was within the standards. Nonetheless, on PET-scan imaging performed 16 months after the treatment onset, multiple hypermetabolic foci persisted: mitral valve, aorta, clavicular and humeral arteries, bypass, popliteal aneurysms, shoulders, bilateral inguinal adenopathies, and a left pulmonary nodule (Table 1 and Figure 1). Thus, we decided to treat the patient with doxycycline and hydroxychloroquine for a total duration of a 24-month period and to monitor serology monthly and PET-scan imaging every 6 months. Regarding clinical efficacy and good tolerance, treatment was extended beyond 24 months. Nevertheless, the patient died in 2019 (still under treatment) because of a complication of his advanced Parkinson’s disease (aspiration pneumoniae on swallowing disorders).

## 3. Discussion

Q fever is a zoonosis transmitted to humans through the inhalation of infected particles released in the environment of sick animals like cattle and sheep. People exposed to contaminated delivery or abortion products through aerosol inhalation or transcutaneous inoculation are at high risk of infection.

Other routes of infection have been described. Human-to-human transmission is exceptional and the role of ticks and food contamination are controversial. Nevertheless, the ability of the bacterium to survive during long periods in the environment make it difficult to respectively identify the source of the infection [5].

Q fever has various clinical manifestations. Almost 60% of infected patients will remain asymptomatic. When symptomatic (40% of cases), acute Q fever mostly presents as a hepatitis and/or a pneumonia [5]. Approximately 1.5–11% of patients will develop focalized persistent infections dominated by cardio-vascular and osteoarticular infections. Persistent focal infections are not exclusive. For example, the association of endocarditis, pulmonary pseudotumor associated with bypass infection, as in our patient.

Endocarditis is the main clinical presentation of persistent Q fever with 60 to 70% to all cases. Patients with a history of valvulopathy and prosthetic cardiac valve have higher risk of endocarditis progression after acute Q fever infection. Echocardiography shows vegetations in only 12–30% of cases. The main finding is a worsening of the valvular function. In our case, *Coxiella burnetii* endocarditis remained possible as the PET-scan showed mitral valve hypermetabolism despite the absence of vegetation during transthoracic echocardiography. The PET-scan has become a central tool in Q fever endocarditis regarding the poor findings of echocardiography. According to Eldin et al., mortality rates among patients with *Coxiella burnetii* endocarditis varied between 5 and 9.3% when diagnosed early and treated correctly [2,5].

Vascular infections due to *C. burnetii* are rare but serious conditions. It is important to distinguish four different entities: aortic vs. non aortic localizations and native vs. prosthetic. Infective native aorta may be more severe because of the risk of aneurysm rupture. Future studies should stratify on these 2 criteria (aortic vs non-aortic, native vs prosthetic). Vascular infections are the second localization of *Coxiella burnetii* persistent focalized infections. After the Netherlands Q fever outbreak, a seroprevalence study performed in patients with aortic or iliac aneurisms showed *C. burnetii* positive serology in 16.7% of cases, among whom 30% were highly suspected of focalized persistent infections. This suggests that Q fever serology should be performed in high-risk patients with vascular conditions in order to screen *C. burnetii* persistent focalized vascular infection and prevent complications that may engage patients vital prognosis [20]. Risk factors are aneurysms and synthetic vascular grafts. Clinical presentation of patients with *C. burnetii* vascular infections is nonspecific. Most of them present with isolated fever and do not report vascular symptoms. Abdominal pain is frequently reported. Inflammatory biological syndrome is frequent, involving high C-reactive protein rates but increase in white blood cell count is rarer [2,5,21,22]. In this case, the only symptom was a four-month recurrent fever. Considering the prolonged fever, the chronic obliterative arterial disease and femoro-popliteal bypass history, the elevated C-reactive protein and the negative blood cultures, we systematically performed *Coxiella burnetii* serology. The diagnosis of focalized persistent *Coxiella burnetii* infection may be overlooked regarding this nonspecific clinical presentation. Thus, physicians need to think of the possibility of Q fever and systematically perform *C. burnetii* serology and specific real-time polymerase chain reactions when afforded.

When there is a suspicion of *C. burnetii* persistent focalized infection, the PET-scan should include sections of the upper and lower limbs, unlike the use of PET-scan for the identification of metastases in cancer that generally only include trunk sections. This is fundamental in order to not dismiss distal mycotic aneurysm or vascular prosthesis infection [22].

Mortality related to cardio-vascular infection drastically decreased over the last decades thanks to doxycycline and hydroxychloroquine prophylaxis and treatment [3,23,24]. Nonetheless, cardio-vascular infection on vascular graft remains a therapeutic challenge because of relapse and vascular ruptures. The mortality rate is much higher in cases of vascular infection than in the case of endocarditis [21] and is even higher in cases of vascular prosthesis infections [25]. Kampschreur et al. compared the mortality rates among Q fever endocarditis and vascular focus infection patients. The case fatality rate was significantly higher in patients with vascular localization compared with endocarditis (18% versus 9.3%, *p* = 0.005) [25]. Obviously, this lethality is highly dependent on early diagnosis, surgery, and clinician expertise (prompt antibiotic therapy with strict monthly monitoring).

Unlike endocarditis, for which surgery is not systematic; treatment is based on both antibiotic and surgical replacement of the infected vascular graft [26,27]. In the case of infected vascular prosthesis, doxycycline (200 mg per day) and hydroxychloroquine (200 mg three times a day) for a 24-month period, and surgery one month after the onset of antibiotics, are recommended [1,2]. Surgical treatment was associated with a better patient and serological outcome. In a retrospective study performed by Eldin et al., mortality at 2.5 years among patients who were treated only with antibiotics was significantly higher than mortality in patients who underwent surgical removal of the infected tissue in addition to antibiotic treatment (28.6% versus 6.5%, *p* = 0.02) [24].

Serological follow-up and drug monitoring are proposed monthly, whereas PET-scan imaging is proposed 24 months after the antibiotic onset [24,26].

In the literature review, fourteen cases of *Coxiella burnetii* vascular graft infections (12 aortic localizations and two non-aortic localizations) were reported with a majority of men (13/15). The mean age was 64 years old. Most had aortic localization of the infection. Antimicrobial therapy combined with surgery were performed in 13 patients. Doxycycline + hydroxychloroquine regimen was used in ten patients and fluoroquinolones were associated with doxycycline in three cases. All patients recovered when antibiotics were associated with surgical removal of the infected lesion. All cases were confirmed with a microbiological criterion (*C. burnetii* IF serology, *C. burnetii* specific PCR) and a lesional criterion on imaging tools (CT-scan and PET-scan). Non-aortic *C. burnetii* vascular graft infections seem to represent a new entity and may differ from aortic localizations in terms of clinical presentation and management. Further studies are needed for a better clinical description (Table 1).

In most patients with acute Q fever (symptomatic primary infection), *Coxiella burnetii* serology become positive 7 to 15 days after symptoms onset, but seroconversion could be delayed in some cases for up to 6 weeks. When symptoms are suspicious of Q fever, serology should be performed systematically. The immunofluorescent assay is the reference method for *Coxiella burnetii* infection diagnosis. The dosage of phase I and II antibodies is necessary in order to distinguish between acute and persistent Q fever.

Phase II IgG ≥1:200 and/or phase II IgM ≥1:50 are consistent with acute Q fever. An increase fourfold in phase II antibodies within a 3 to 6 week interval is considered significant to retain the diagnosis. Antibodies kinetics showed a decrease in titles within three to six months. In some patients, phase II IgG remain detectable lifelong.

Regarding persistent focalized infection, phase I IgG titles are superior to phase II IgG. A cut-off of phase I IgG ≥1:800 is highly evocative of persistent Q fever. Frankel et al. described that phase I IgG ≥1:6400 is associated with *C. burnetii* endocarditis with a positive predictive value of 75% [28]. However, lower titles of phase I Ig G have been described in patients with confirmed Q fever endocarditis [5].

Molecular tools are widely used for Q fever diagnosis. Compared with conventional PCR, qPCR (quantitative PCR) is easy, simple, and fast. This technique is also sensitive and can detect 10 to 100 bacteria in serum or blood. In the French Reference Center for Rickettioses, Q Fever, and Bartonelloses, Marseille, France, Aix-Marseille Université, PCR sensitivity was increased with clinical sample lyophilization (detection threshold evaluated at one bacterium).

When available, qPCR is positive in the serum of patients with acute Q fever during the first two weeks of the infection before the seroconversion. This is a precious resource to detect the infection as soon as possible for the early treatment onset in order to prevent persistent focalized infection evolution. Another advantage of qPCR is the quantification of the bacterial inoculum, which is predictive of persistent infection, especially in patients at risk [5]. In patients with *C. burnetii* vascular graft infections, qPCR and/or culture of the vascular tissue or the graft material is a major criterion for diagnosis confirmation (Table 1). Fluorescence in situ hybridization has recently been proposed as a promising and innovative diagnosis tool for the diagnosis of *C. burnetii* focalized infection, although its sensitivity seems to be superior in endocarditis than in vascular infection [29,30].

In the present case, blood PCR was negative and the diagnosis of *Coxiella burnetii* femoro-popliteal bypass infection was based on the presence of a positive serology and the presence of hypermetabolism, on the PET-scan imaging [30,31]. PET-scan has moved from being a fundamental technique describing cell metabolism to an essential tool in clinical practice. According to Hagenaars et al., PET-scan has a positive predictive value of 78% and a negative predictive value of 77% for the diagnosis of Q fever vascular infections [31]. In a retrospective study involving 167 patients with active *C. burnetii* infection, ^18^F-FDG PET/CT changed the diagnosis for 62.6% of patients and revealed a previously unsuspected focus of infection in 38.7% of cases. This innovation was a strong argument for a paradigm change from chronic Q fever to *C. burnetii* persistent focalized infection. The ancient term “chronic Q fever” was regrouping entities that differed in term of clinical presentation, prophylaxis, and treatment strategies. *C. burnetii* persistent focalized infection is a recent concept resulting from the introduction of the PET-scan for the management of *C. burnetii* infected patients, which made possible the description of new Q fever entities. It is more accurate as it enables a specific diagnosis of the infected organ, which leads to better patient care [32].

PET-scan has become a central tool to detect persistent *C. burnetii* focalized infections [32]. It is now also a central tool to detect relapses, complications, and manage the duration of the antibiotic therapy and guide surgery [33,34].

Regarding other infections on the vascular prosthetic material reported, Maarten A.J. van de Weijer et al. described in a systemic review that graft infections occurred in 2.4% of patients who underwent femoro-popliteal bypass surgery within the last 30 days [35]. The most common causative bacteria are *Staphylococcus aureus*, followed by coagulase negative *Staphylococci* and Gram-negative bacilli [36].

In contrast to extracellular bacteria that may infect vascular grafts, *C. burnetii* is able to survive and multiply within an intracytoplasmic acidic vacuole, the containing *Coxiella* vacuola (CCV), in which the bacterium may escape the immune system for years, being in a dormant stage, as reported for *M. tuberculosis* [37,38]. Thus, Q fever infection of prosthetic vessels is a new challenge for physicians since the penetration of antibiotics in the prosthetic material is known to be low [39,40].

Q fever represents a silent but global public health problem since it causes outbreaks in animals and humans. The evolution to a persistent focalized infection is a major threat because of the diagnosis delay usually occurring at the complications stage. When *C. burnetii* causes coxiellosis in domestic ruminants, the whole herd can quickly be affected by the disease, causing economic issues. For instance, a loss of EUR 307 million was estimated during the outbreak in the Netherlands [41]. Q fever represents a One Health problem as it impacts human, environment, and animal health. Prevention of the infection based on these three axes seems to be the most effective way to reduce Q fever complications and the evolution to persistent localizations. Serological and/or molecular screening among reservoirs, vaccination, outbreak control, environment management, early diagnosis, and treatment of human cases and transdisciplinary collaboration are some measures that should be implemented in a One Health approach in order to decrease Q fever incidence [42,43].

Among patients with acute Q fever, hydroxychloroquine should be added if there is a high risk of progression to focalized persistent infection such as vascular graft and prosthesis [5,30,44]. Patients with risk factors of Q fever vascular infections (aneurisms, vascular graft, prosthesis…) should be systematically screened using *Coxiella burnetii* serology and if positive, the PET-scan imaging is the tool of choice to identify hypermetabolic foci most likely resulting from *C. burnetii* persistent infection [5,25].

## 4. Conclusions

Persistent focalized Q fever should be considered in patients with persistent fever history, especially if they have risk factors such as vascular aneurysms and/or vascular grafts. The increasing use of vascular prostheses should raise the awareness of *C. burnetii* vascular infections in prosthetic material. PET-scan imaging has become an essential tool to detect these vascular infections for which the diagnosis is based on one microbiological and one lesional criterion. Doxycycline and hydroxychloroquine, in addition to the removal of the infected material, is the treatment of choice, and close clinical, serological, and radiological monitoring could help to detect relapses and complications. A one health approach should be used in order to prevent human transmission from animal reservoirs and contaminated environments. Screening and early treatment of acute *Coxiella burnetii* infection has been shown to be effective in preventing the evolution to focalized persistent infections and complications.

## Figures and Tables

**Table 1:** Cases of reported *Coxiella burnetii* vascular graft infections.

Cases	Sex/Age	*C. burnetii* Infection Documentation (Serology, PCR, Culture…)	Vascular Infection Localization (Imaging Tool)	Treatment (Antibiotherapy and Surgery)	Outcome (at Time of Publication)	**Country/Ref**
**Aortic vascular prosthesis infection**						
1	Man/78 years old	-Serology (IF ^1^) Phase I: IgG: 1:25,600, IgM: 1:400, Phase II IgG: 1:25,600, IgM: 1:400. -Positive PCR on serum	Abdominal under-renal aortic graft (TDM, PET-scan)	-DH ^2^ for 18 months -Surgery after 3 months	Recovery (three years follow-up)	France [6]
2	Man/63 years old	-Positive 16S rRNA PCR + sequencing on fragment of the para-iliac mass -Serology (IF) IgGI: 1:1,600, IgGII: 1:3,200	Dacron aorto-bifemoral graft (CT-scan)	-partial removal of the prosthetic graft -DH	after 18 months: -Asymptomatic -IgG I:1600 and IgGII: 3200	Switzerland [7]
3	Man/66 years old	-Complement Fixation Assay: CFT phase I antibody Titer: 1:1,280; CFT phase II antibody titer: 1:1,280	Aorto-bifemoral bypass (MRI, CT-scan)	-oral doxycycline (200 mg/d) and ciprofloxacin (1000 mg/d) -graft excision	Recovery (21 months follow-up)	United Kingdom [8]
4	Man/70 years old	-Serology (IF) IgGI: 1:25,600; IgAI: 1:3200; IgGII: 1:51,200; IgAII: 1:6,400 -Positive real-time PCR (IS30a sequence) on a fragment of the aortic mass and the puncture liquid	Aortobiiliac endoprosthesis (CT-scan, PET-scan)	-DH -surgery three months later	Recovery (six months follow-up)	France [9]
5	Man/67 years old	-Serology (IF) IgGI: 1:800, IgMI: 0, IgAI: 1:800, IgGII: 1:1,600, IgMII: 0, IgAII: 1:800 -Positive aortic graft culture	Pseudoaneurysm of an aortic graft (CT-scan, MRI)	Doxycycline 200 mg/day and ofloxacine 600 mg/day Surgical excision of the aortic graft three weeks later	Recovery (three years follow-up)	France [10]
6	Man/46 years old	-Serology: IgGI: 1:51,200, IgMI: 1:25, IgAI: 1:200, IgGII: 1:102,400, IgMII: 1:50, IgAII: 1:400 -Positive specific PCR in sternal effusion	Aortic vascular graft (Bentall) (CT-scan, PET-scan)	DH	Recovery (21 months follow-up)	France [11]
7	Man/58 years old	Serology IgGI: 1:10,000	Abdominal aortic prosthetic graft (FDG-PET-scan)	Antibiotics (not detailed) Surgical graft removal	Recovery (9 months follow-up)	The Netherlands [12]
8	Man/67 years old	-Serology IgGI: 1:2,048, IgGII: 1:1,024, IgM: 0 -Positive PCR on vascular tissue (post mortem)	Perirenal abdominal aortic graft (CT angiogram, MRI)	DH Surgical repair of abdominal aortic aneurysm and bilateral renal artery reconstruction	Died from acute pulmonary embolism 22 months since admission	Canada [13]
9	Woman/69 years old	-Positive panbacterial PCR on interlaminar L3-L4 biopsy and purulent fluid. -Serology: IgGI: 1:4,096, IgMI > 1:2,048 IgGII: 1:16,348, IgMII: 1:1,024	Aortic endoprosthesis (CT-scan, MRI, PET-scan)	DH Aortic endoprosthesis removal	Recovery (5 months follow-up)	Belgium [14]
10	Man/62 years old	-Positive specific PCR on blood, thrombus and tissue culture -Serology IgGI: 1:2,048, IgGII: 1:4,096	Infrarenal aortic bifurcated endograft (CT-scan)	Doxycycline 200 mg/day and cirprofloxacin 1500 mg/day abdominal wall of aneurysmal sac (including abscesses) removal endograft left in place	Recovery (six months follow-up)	The Netherlands [15]
11	Woman/55 years old	-Serology IgGI: 1:4,096, IgII: 1:4,096 -Positive specific PCR on blood, fluid material and prosthesis	Supracoronary tube graft repair of the ascending thoracic aorta (CT-scan, FDG PET-scan)	DH One year later (non-responsiveness to conservative treatment), aortic graft removal + Moxifloxacin	Recovery (six months follow-up)	The Netherlands [16]
12	Man/71 years old	-Serology IgGI: 1:8,192, IgGII: 1:8,192 -Positive specific real-time PCR on graft material	Aorto-bi-iliac graft (PET-scan)	DH Graft replacement and cure of the false aneurysm	Not mentioned	Switzerland [17]
**Non-aortic vascular prosthesis infection**						
13	Man/61 years old	-Serology Phase I: IgG: 1:6,400, IgM: 1:50, IgA: 0 Phase II: IgG: 1:6,400, IgM: 0, IgAII: 0 -Positive 16S rRNA PCR+ sequencing and positive specific PCR (real-time PCR of the regions IS1111 and IS630) on periprosthetic biopsies and vascular arteriovenous grafts	Left humeral-axillary arteriovenous graft (CT-scan with tagged white blood cells of the left arm)	-DH -Surgery: partial removal of the infected prosthetic AV grafts	Recovery (undergoing treatment)	Spain [18]
14	Woman/50 years old	-Serology (IF) IgGI: 1:3,200, IgAI: 1:1,400, IgMI: 0 IgGII: 1:1,600, IgAII: 1:1,400, IgMII: 0 -Positive 16S rRNA PCR in the prosthetic valvular material -Positive specific PCR of the regions IS1111 and IS30a in the blood and vascular graft	Hemodialysis vascular graft (PET-scan)	Complete surgical removal of the infected vascular graft DH	Recovery (three months follow-up)	France [19]
15	Man/81 years old	-Serology (IF) IgGI: 1:800, IgMI: 0, IgAI: 1:100 IgGII: 1:200, IgMII: 0, IgAII: 0.	Femoro-popliteal bypass (PET-scan)	DH	-Clinical improvement -multiple hypermetabolisc foci persistence	Our case

^1^ IF: immunofluorescence assay; ^2^ DH: doxycycline and hydroxychloroquine.

## Data Availability

Data is contained within the article.

## References

[1] Wegdam-Blans M., Vainas T., van Sambeek M.R., Cuypers P., Tjhie H., van Straten A., Teijink J. (2011). Vascular Complications of Q-fever Infections. Eur. J. Vasc. Endovasc. Surg..

[2] Maurin M., Raoult D. (1999). Q Fever. Clin. Microbiol. Rev..

[3] Million M., Walter G., Thuny F., Habib G., Raoult D. (2013). Evolution from Acute Q Fever to Endocarditis Is Associated With Underlying Valvulopathy and Age and Can Be Prevented by Prolonged Antibiotic Treatment. Clin. Infect. Dis..

[4] Fournier P., Casalta J., Piquet P., Tournigand P., Branchereau A., Raoult D. (1998). *Coxiella burnetii* Infection of Aneurysms or Vascular Grafts: Report of Seven Cases and Review. Clin. Infect. Dis..

[5] Eldin C., Mélenotte C., Mediannikov O., Ghigo E., Million M., Edouard S., Mege J.-L., Maurin M., Raoult D. (2017). From Q Fever to *Coxiella burnetii* Infection: A Paradigm Change. Clin. Microbiol. Rev..

[6] Hamon A., El Sayed F., Bouchand F., Davido B., Duran C., Coggia M., Javerliat I., Dinh A. (2020). Infection d’endoprothèse aortique à *Coxiella burnetii*. Med. Mal. Infect..

[7] Senn L., Franciolli M., Raoult D., Moulin A., Von Segesser L., Calandra T., Greub G. (2005). *Coxiella burnetii* vascular graft infection. BMC Infect. Dis..

[8] O’Donnell M.E., Manshani N., McCaughey C., Soong C., Lee B. (2007). *Coxiella burnetii* infection of an aortic graft with multiple vertebral body erosion. J. Vasc. Surg..

[9] Merhej V., Cammilleri S., Piquet P., Casalta J.-P., Raoult D. (2012). Relevance of the positron emission tomography in the diagnosis of vascular graft infection with *Coxiella burnetii*. Comp. Immunol. Microbiol. Infect. Dis..

[10] Piquet P., Raoult D., Tranier P., Mercier C. (1994). *Coxiella burnetii* infection of pseudoaneurysm of an aortic bypass graft with contiguous vertebral osteomyelitis. J. Vasc. Surg..

[11] Dutasta F., Richaud C., Michon A., Ragone E., Podglajen I., Mainardi J.-L. (2016). Use of 18F-FDG PET/CT for diagnosis of vascular graft infection with spread to sternum caused by *Coxiella burnetii*. Infect. Dis..

[12] van Assen S., Houwerzijl E.J., van den Dungen J.J., Koopmans K.-P. (2007). Vascular graft infection due to chronic Q fever diagnosed with fusion positron emission tomography/computed tomography. J. Vasc. Surg..

[13] Stokes W., Janvier J., Vaughan S. (2016). Chronic Q Fever in Alberta: A Case of *Coxiella burnetii* Mycotic Aneurysm and Concomitant Vertebral Osteomyelitis. Can. J. Infect. Dis. Med Microbiol..

[14] Waelbers V., Desmet S., De Munter P., Van Loon J., Fourneau I. (2020). Vertebral Osteomyelitis or Infected Abdominal Aortic Endograft? A Rare Case of Q Fever. Ann. Vasc. Surg..

[15] Kloppenburg G.T., van de Pavoordt E.D., de Vries J.-P.P. (2011). Endograft-preserving therapy of a patient with *Coxiella burnetii*-infected abdominal aortic aneurysm: A case report. J. Med. Case Rep..

[16] Wegdam-Blans M.C., ter Woorst J.F., Klompenhouwer E.G., Teijink J.A. (2012). David procedure during a reoperation for ongoing chronic Q fever infection of an ascending aortic prosthesis. Eur. J. Cardio-Thoracic. Surg..

[17] Dvorak S., Bizzini A. (2020). *Streptococcus anginosus* and *Coxiella burnetii* vascular graft co-infection. IDCases.

[18] Vecchio M.G.-D., Vena A., Valerio M., Marin M., Verde E., Muñóz P., Bouza E. (2014). *Coxiella burnetii* Infection in Hemodialysis and Other Vascular Grafts. Medicine.

[19] Ernest V., Cammilleri S., Amabile P., Fedi M., Burtey S., Von Kotze C., Pelletier M., Moal V., Guedj E., Perron C. (2018). Hemodialysis vascular graft as a focus of persistent Q fever. Infection.

[20] Hagenaars J.C., Wever P.C., van Petersen A.S., Lestrade P.J., de Jager-Leclercq M.G., Hermans M.H., Moll F.L., Koning O.H., Renders N.H. (2014). Estimated prevalence of chronic Q fever among Coxiella burnetii seropositive patients with an abdominal aortic/iliac aneurysm or aorto-iliac reconstruction after a large Dutch Q fever outbreak. J. Infect..

[21] Melenotte C., Protopopescu C., Million M., Edouard S., Carrieri M.P., Eldin C., Angelakis E., Djossou F., Bardin N., Fournier P.-E. (2018). Clinical Features and Complications of *Coxiella burnetii* Infections from the French National Reference Center for Q Fever. JAMA Netw. Open.

[22] Million M., Raoult D. (2015). Recent advances in the study of Q fever epidemiology, diagnosis and management. J. Infect..

[23] Fenollar F., Fournier P., Carrieri M.P., Habib G., Messana T., Raoult D. (2001). Risks Factors and Prevention of Q Fever Endocarditis. Clin. Infect. Dis..

[24] Eldin C., Mailhe M., Lions C., Carrieri P., Safi H., Brouqui P., Raoult D. (2016). Treatment and Prophylactic Strategy for Coxiella burnetii Infection of Aneurysms and Vascular Grafts: A Retrospective Cohort Study. Medicine.

[25] Kampschreur L.M., Delsing C.E., Groenwold R.H.H., Wegdam-Blans M.C.A., Bleeker-Rovers C.P., de Jager-Leclercq M.G.L., Hoepelman A.I.M., van Kasteren M.E., Buijs J., Renders N.H.M. (2014). Chronic Q Fever in the Netherlands 5 Years after the Start of the Q Fever Epidemic: Results from the Dutch Chronic Q Fever Database. J. Clin. Microbiol..

[26] Botelho-Nevers E., Fournier P.-E., Richet H., Fenollar F., Lepidi H., Foucault C., Branchereau A., Piquet P., Maurin M., Raoult D. (2007). Coxiella burnetii infection of aortic aneurysms or vascular grafts: Report of 30 new cases and evaluation of outcome. Eur. J. Clin. Microbiol. Infect. Dis..

[27] Million M., Thuny F., Richet H., Raoult D. (2010). Long-term outcome of Q fever endocarditis: A 26-year personal survey. Lancet Infect. Dis..

[28] Frankel D., Richet H., Renvoisé A., Raoult D. Q Fever in France, 1985–2009—Volume 17, Number 3—March 2011—Emerging Infectious Diseases Journal—CDC. https://wwwnc.cdc.gov/eid/article/17/3/10-0882_article.

[29] Prudent E., Lepidi H., Angelakis E., Raoult D. (2018). Fluorescence In Situ Hybridization (FISH) and Peptide Nucleic Acid Probe-Based FISH for Diagnosis of Q Fever Endocarditis and Vascular Infections. J. Clin. Microbiol..

[30] Melenotte C., Million M., Raoult D. (2020). New insights in *Coxiella burnetii* infection: Diagnosis and therapeutic update. Expert Rev. Anti-Infect. Ther..

[31] Hagenaars J.C., Wever P.C., Vlake A.W., Renders N.H.M., Van Petersen A.S., Hilbink M., De Jager-Leclercq M.G.L., Moll F.L., Koning O.H.J., Hoekstra C.J. (2016). Value of ^18^F-FDG PET/CT in diagnosing chronic Q fever in patients with central vascular disease. Neth. J. Med..

[32] Eldin C., Melenotte C., Million M., Cammilleri S., Sotto A., Elsendoorn A., Thuny F., Lepidi H., Roblot F., Weitten T. (2016). ^18^F-FDG PET/CT as a central tool in the shift from chronic Q fever to Coxiella burnetii persistent focalized infection: A consecutive case series. Medicine.

[33] Melenotte C., Million M., Hartung O., Botelho-Nevers E., Claudel M., Craighero F., Brouqui P., Raoult D. (2012). Query rectal bleeding. Lancet.

[34] Melenotte C., Million M., Audoly G., Gorse A., Dutronc H., Roland G., Dekel M., Moreno A., Cammilleri S., Carrieri M.P. (2016). B-cell non-Hodgkin lymphoma linked to Coxiella burnetii. Blood.

[35] van de Weijer M.A., Kruse R.R., Schamp K., Zeebregts C.J., Reijnen M.M. (2015). Morbidity of femoropopliteal bypass surgery. Semin. Vasc. Surg..

[36] Szilagyi D.E., Smith R.F., Elliott J.P., Vrandecic M.P. (1972). Infection in Arterial Reconstruction with Synthetic Grafts. Ann. Surg..

[37] Mege J.-L., Maurin M., Capo C., Raoult D. (1997). *Coxiella burnetii*: The ‘query’ fever bacterium: A model of immune subversion by a strictly intracellular microorganism. FEMS Microbiol. Rev..

[38] Raoult D., Marrie T., Mege J. (2005). Natural history and pathophysiology of Q fever. Lancet Infect. Dis..

[39] Revest M., Camou F., Senneville E., Caillon J., Laurent F., Calvet B., Feugier P., Batt M., Chidiac C. (2015). Medical treatment of prosthetic vascular graft infections: Review of the literature and proposals of a Working Group. Int. J. Antimicrob. Agents.

[40] Mandell J.B., Orr S., Koch J., Nourie B., Ma D., Bonar D.D., Shah N., Urish K.L. (2019). Large variations in clinical antibiotic activity against *Staphylococcus aureus* biofilms of periprosthetic joint infection isolates. J. Orthop. Res..

[41] van Asseldonk M., Prins J., Bergevoet R. (2013). Economic assessment of Q fever in the Netherlands. Prev. Vet. Med..

[42] Rahaman R., Milazzo A., Marshall H., Bi P. (2019). Is a One Health Approach Utilized for Q Fever Control? A Comprehensive Literature Review. Int. J. Environ. Res. Public Health.

[43] Espí A., del Cerro A., Oleaga Á., Rodríguez-Pérez M., López C.M., Hurtado A., Rodríguez-Martínez L.D., Barandika J.F., García-Pérez A.L. (2021). One Health Approach: An Overview of Q Fever in Livestock, Wildlife and Humans in Asturias (Northwestern Spain). Animals.

[44] Million M., Walter G., Bardin N., Camoin L., Giorgi R., Bongrand P., Gouriet F., Casalta J.-P., Thuny F., Habib G. (2013). Immunoglobulin G Anticardiolipin Antibodies and Progression to Q Fever Endocarditis. Clin. Infect. Dis..

